# Hospital and Institutional News

**Published:** 1914-04-11

**Authors:** 


### HOSPITAL AND INSTITUTIONAL NEWS.

### OUR PRIZE SCHEME FOR EQUIPMENT "TIPS."

Those who turn as their chief interest to the *Institutional Worker* section of The Hospital will note with interest the brisk correspondence which has been running for the last few weeks on the question of the best method of filing out-patient case-papers. Many interesting points have been cleared up, and the exchange of criticism has been as entertaining as it has been frank and outspoken. No less interest, however, is attaching on the part of other readers to the Prize Scheme (see page 55) for a description of the best ward-locker in actual use, or of a suggested improvement, which shall reach us on or before April 18. The readers of The Hospital will be the judges, as the prizes will go to those whose description secures the greatest number of votes. Those who like to make two, three, or five guineas, which is the amount of the prizes offered, will have an opportunity of competing during the Easter holidays, and cannot do better than send to us a description of a ward-locker, which is the article chosen for a start, as being one for the first-class design of which there is a large demand, in spite of the many patterns on the market. We know from personal inspection of institutions over many years, however, that some of the really best types of furniture or appliances are the home-made articles, designed by individual officers for merely local use, and the object of the Prize Scheme is to offer a sufficient money inducement to unearth these "tips" of equipment, or expense- and labour-saving devices, for the mutual benefit of all the institutions. To ensure also that the subjects of the prizes may be of the most wanted type, it remains only for those officers or institutions *in need* of some requirement also to let us know, so that every article for which there is a demand, or in which an improvement is sought, may be promptly offered for competitive description.

### A MENTAL HOSPITAL STAFF ON STRIKE.

Owing to the Easter holidays, which compel us to go to press earlier than usual, we are not able this week to publish a complete account of the short-lived strike in which about twenty of the male attendants at the County Mental Hospital, Rainhill, attempted to indulge. It appears that, owing to porridge being provided in place of meat at breakfast, these attendants refused to eat the food, and then declined to go on duty. It is stated that the National Asylum Workers' Union—the new organisation, which is not to be confused with the Asylum Workers' Association, of which Dr. Shuttleworth is the secretary—intervened in the matter, and that peace was not restored until Dr. Thomas Philip Cowen, the medical superintendent, appeared on the scene, and dealt with it promptly by arranging for a return to the old breakfast diet until the mental hospital committee could meet and dispose of the alleged grievances. We have dealt before now with the unthinkable set-back to the whole mental hospital attendants' position and status which an attempted "strike" would cause, and are sure that the men themselves will learn to regard with bitter regret any organised or individual attempt pointing in this direction.

### THE HOSPITAL POSITION IN ULSTER.

A meeting has been held in London to consider the most practical means of supporting the auxiliary ambulance and hospitals which have been established in the Province of Ulster to meet the possible emergency of civil war. The Marchioness of Londonderry pointed out that many houses had been converted into hospitals, and appealed for help for the poorer districts which had not yet been able to provide hospital accommodation and necessaries. Mr. Edmond Owen emphasised this view by remarking that the outlying districts of Ulster in particular were deficient in *x*-ray apparatus, operating theatres, drugs, and hospital stores. Where ten or twenty beds had been provided, as many as a hundred might be required if trouble arose. We suspect that it may be more difficult to supply deficiencies of equipment than deficiency of *personnel*, since there is a spirit of adventure to be gratified by volunteer service which is not assuaged by the drawing of a cheque or the despatching of supplies. It is understood that, considering the difficulties, preparations in case of need have been surprisingly forward for some time.

### A GENERAL POST AMONG LONDON HOSPITAL SECRETARIES.

Mr. Gilbert G. Panter, formerly assistant secretary at the Great Northern Central Hospital, whose appointment as secretary to the Belgrave Hospital for Children, in succession to Mr. Gregg, was announced by us last week, has since resigned that post to take up the secretaryship of the Great Northern Central Hospital, where a vacancy now occurs. Mr. Lewis H. Glenton-Kerr, whom he succeeds, was appointed in 1894, when he followed Mr. William T. Grant. In these twenty years the number of beds at the Great Northern Central Hospital has risen from seventy-two to 180. Mr. Gilbert Panter has thus received a very rapid promotion, for to rise from assistant secretary to secretary of a big London hospital without intermediate experience elsewhere is a feat upon which his good fortune as well as his abilities must divide the honours. The new assistant secretary at the Great Northern is Mr. J. H. Cowper, who has been for two years chief clerk at the Hampstead General Hospital, previous to which he had a post at Queen Charlotte's.

### THE STAFF DIFFICULTY IN INFECTIOUS HOSPITALS.

Dr. Cuff, principal medical officer of the Metropolitan Asylums Board, who was instructed last November to report on the conditions offered to the nursing staff in the Board's infectious hospitals, and to offer any suggestions he might have to make the service more attractive, has issued his statement, which, together with one prepared by a sub-committee, was presented last Saturday. The difficulty of obtaining and retaining nurses for these institutions has long been known, and we draw attention to it here because of the light which Dr. Cuff's suggestions throw on another aspect of the question. He points out that the difficulty is due to fever nursing being virtually a blind-alley profession; to its monotony, isolation, the attraction of other professions now open to women of moderately good education; to its poor pay; to emigration; and to the fact that the Metropolitan Asylums Board is a Poor-Law authority. He therefore recommends tapping a larger source of supply, by lowering the minimum age-limit for probationers from 21 to 19, and making the service more attractive, in which direction the sub-committee alluded to has submitted a slightly increased scale of salaries, working out roughly as a rise of £2 per annum all round. Their other suggestions include an extra whole day's or night's leave each month for day and night nurses respectively, an increase in the annual leave after three years' service, and certain diet improvements. Again, on the report of Dr. J. W. Mullen, medical superintendent of Ladyhill Infectious Hospital, Salford, the Health Committee of Salford have decided to advance the salaries of probationer nurses by £2, £3, and £4 in their respective years. We touch on these instances as a symptom of the dissatisfaction which is being manifested by the staffs of this class of institution, now apparent by the formation of an Association to promote the interests of the assistant medical officers in the fever, children's, and tuberculosis services of the Metropolitan Asylums Board. The honorary secretary of the new Association is Dr. E. L. Goffe, North-Eastern Hospital, Tottenham.

### COMBINATION IN THE FEVER HOSPITAL SERVICE.

The new Association has not had time yet to complete its plan of campaign, but when this is ready it will be instructive to compare it with that of the recent Association of Assistant Medical Officers in Mental Hospitals, whose aims and development have been frequently discussed in our columns. It is at once obvious that the two services have much in common, of which active and ambitious men most complain. The mental hospital and the infectious hospital are both means of shutting men off from their fellows. The chances of promotion in both are far from rosy; the amount of routine work, and the lack of interchange of technical knowledge within and without the service is a serious drawback, while the scale of salaries is not such as to excite men to stay in them and push to the front. It is also interesting to compare the way in which the two sexes attempt to remedy their position and prospects. The nurses are slow to come into, or soon leave, the service, which is a purely negative proceeding, that can only be successful where, as in this case, other professions are competing for their services in sufficient numbers to make their scarcity officially felt. It is a sort of passive strike method, but most fortunately without the waste and rancour of what trade unionists call "direct action"—a proceeding unthinkable in the hospital world. The men, not content with staying away, though negative pressure of a similar kind is being exerted in most resident medical posts to-day, deliberately resort to combination. As we have previously pointed out, this principle has done much for attendants in mental hospitals, for whom the virtual ban on marriage, under which the assistant medical officers still groan, has been practically removed—it is not surprising, therefore, that the medical staff should also resort to it.

### A HOSPITAL MATRON'S FINANCE.

That women, and matrons, can prove on occasion able hospital financiers is seen by the success which has attended the praiseworthy energies of Miss Kate L. Ray, matron of St. Mary's Hospital for Women and Children, Plaistow. Four years ago she started a Million Pennies Fund to provide a new nurses' home at the institution, where one is certainly badly needed. Of this sum she has successfully raised rather more than three-quarters, or about £3,340. With the means at her disposal, largely such as unaided personal enthusiasm can initiate, no hospital secretary could be anything but proud of the result. Does this point to women one day seeking to add to their achievements in nursing the honours also of hospital superintendentship?

### THE FIRST LEGACY FOR FOURTEEN YEARS.

The late Mr. T. E. Crocker, who died on March 8, has left by will a legacy of £500 to the Nelson Hospital, Wimbledon, free of legacy duty. Mr. Crocker made a handsome contribution to the new building fund during his lifetime. This legacy will be the first legacy received by the hospital since its foundation in 1900. This very astounding succession of lean years and testamentary neglect has at last been broken, and we congratulate Mr. Kirkaldy, the secretary, and the board upon this fact. Perhaps the tide will now set in the hospital's direction.

### WAKING UP THE ANNUAL MEETING.

Hospitals fall into two classes in the treatment of the annual meeting. Those, and they are the large majority, who frankly give it up as a bad job, take the report as read, and would take the meeting as held too if it were not a necessary part of constitutional routine; and those who feel that something must be done to relieve the intolerable tedium of this annual function. The latter make various and usually spasmodic efforts to lure the attention of the governors and their friends by cinematograph slides, or, in the provinces, by a procession in which Territorials, boy scouts, or the local fire brigade are often induced to take part. Warneford Hospital, Leamington, has adopted the idea of a procession for the first time this year, taking example, no doubt, from Hereford, and the collections in the church, for a service was held, and the sale of programmes in the streets *en route* realised in round figures £143. That is so much to the good, for which the Warneford Hospital must be congratulated. But while we have always preached the doctrine that the annual meeting should be made a very prominent and successful function, and thereby welcome any attempt to secure attention to it, we cannot help feeling that there exists a more excellent way than processions or cinematograph slides. These have the genuine defect of being adjuncts: their purpose is to gild the pill of the annual meeting. We believe that the annual meeting itself can and should be made the centre of interest.

### THE ANNUAL MEETING AS AN EVENT IN ITSELF.

One way to do this is to make it a public function by having someone who possesses authoritative knowledge of hospitals to come down and give an address either from the chair or in support of the adoption of the report. Lord Northcliffe did this with marked success at Coventry a few weeks ago, and Leicester Infirmary always makes its annual meeting one of the chief county events of the year by similar means. To invite mere figureheads, even if they be presidents or vice-presidents, is of no value. Bishops, Lord-Lieutenants, peers of the realm are useless. Men of position, who are recognised to have authoritative knowledge of hospitals, are required—one such as we all know happens to belong to the peerage. The presence of such men will serve to stir up interest in all that the voluntary hospital system stands for, and thereby make each particular hospital a centre radiating personal service throughout the whole district which it serves. Processions, amusements in any form, will never do this, but to do it is the true function of every annual meeting.

### THE LATE MR. BRUCE CLARKE.

Everyone who has been connected with St. Bartholomew's Hospital during the last forty years will have heard with regret of the death of Mr. W. Bruce Clarke, until quite lately senior surgeon to that institution. Mr. Bruce Clarke, who was sixty-four years of age, died on March 28 of pneumonia, following influenza, at Eastbourne, where he had gone to live since his retirement from active work. He was an Oxford University graduate, as well as a Fellow of the Royal College of Surgeons, where he was a member of the Council. One of his marked characteristics was a constant endeavour to keep fully abreast of the best new ideas in surgery; indeed, he was unwearied in going to see for himself what other surgeons were doing, and always ready to recognise the value of other men's work, even though they might be younger than himself. A man of wide culture and a thoroughly humane disposition, he enjoyed a wide popularity with his colleagues, his students, and his patients, both in the wards of St. Bartholomew's and outside. He was at one time also on the surgical staffs of St. Peter's Hospital for Stone and of the West London Hospital. Mr. Bruce Clarke was twice married.

### RETROGRADE PROPOSAL BY THE DENTAL PROFESSION.

An extraordinary suggestion in relation to the dental profession was recently made at a meeting of the West of Scotland Branch of the Incorporated Dental Society. The suggestion was neither more nor less than that dental surgeons should in future be freed from the control of the General Medical Council and that students should not be required to attend the medical portion of their curriculum. The meeting at which this suggestion was brought out was one which dealt with the prospect of the formation of a national dental service, the idea being that sooner or later dental insurance benefits will be offered to supplement the medical benefits of the Insurance Act. It may well be wondered if the West of Scotland Branch of the Incorporated Dental Society has not developed a completely warped notion of the interests of the dental profession. That such a suggestion was discussed seems little less extraordinary than the tenor of the proposal itself. Surely the true direction of advance should be towards the ideal of making it compulsory for every dentist to take a full medical qualification. If dental surgery and oral hygiene are not special branches of general medicine, it is difficult indeed to realise their position. It is sufficiently obvious that the profession of dentistry stands in need of improved status to-day and not of divorcement from the medical profession.

### VISITING DENTISTS FOR POOR-LAW INFIRMARIES.

In our issue of January 24, 1914, we published an interesting paper dealing with the importance of preventing oral sepsis in connection with the prophylaxis and treatment of tuberculosis. The letter from "A Sanatorium Officer" in our last number shows that the necessity for making provision in this respect is forcing itself upon the attention of sanatorium officials. The writer of the letter asks pertinently if the time will ever come when the State Insurance will include dental benefit. It is, therefore, an opportune moment to inquire what provision is being made under this heading in the other State Department which deals with medical treatment—the Poor-Law Service. The large Poor-Law schools and many of the Guardians' receiving homes for children have visiting dentists, but it is surprising to find that practically no provison has been made in the Poor-Law infirmaries. Out of fifteen of the chief London infirmaries at which inquiries were made recently in only one of them is a dentist employed, whilst at one other the matter is now under discussion by the Guardians. Practically the only dental work carried out in these infirmaries consists of extractions, and these are performed by the medical staff. Yet the importance of the cleanliness of the mouth in connection with operations upon it and the alimentary canal and the association of oral sepsis with rheumatoid arthritis, anæmia, gastric troubles, and so on are universally recognised. Medical men are only too familiar with the immense amount of dental disease existing amongst the poor of our large towns, and with the dire effects such neglect has upon the general health. There can surely be no doubt that every Poor-Law infirmary should have a visiting dentist amongst its staff.

### THE OLD MANCHESTER ROYAL INFIRMARY SITE.

We have often alluded to the discussions in Manchester which have arisen over the proper way to dispose of the old infirmary site in Piccadilly, and in which proposals for a municipal art gallery and for a business exchange have found vigorous and opposed supporters. Among the advocates of the municipal art gallery suggestion was the late Mr. James Gresham, a wealthy man, and director of the firm of engineers which bears his name, whose will and estate, amounting to over £400,000, have now been published. After leaving £500 to the Manchester Royal Infirmary, and similar amounts to the Salford Blind Hospital, the Old Trafford Blind Asylum, and the Old Trafford Deaf and Dumb School, he bequeathed, subject to his wife's life interest, such of his oil and water-colour pictures, not his own work, as his trustees may select to be handed over to the Art Gallery Committee of the Manchester Corporation, on the proviso that a municipal art gallery shall be built on the infirmary site in Piccadilly within five years of his death. Will this offer prove tempting enough to decide the question over which so much time has been spent, and so many words wasted, with so little result?

### MR. WALTER CARNT'S RECOVERY.

It is not only the personal friends of Mr. Walter G. Carnt, general superintendent of Manchester Royal Infirmary, who will learn with pleasure of his sure but slow recovery from the operation which he underwent in February last. The operation, the necessity for which had been foreseen for some time, was serious enough to keep him in bed rather more than a month, but before the end of March he was able to be sent away to the South of England, and, if his present rate of progress be maintained, he should be able to return to work by about the middle of May.

### THE INSURANCE ACT AND HOSPITAL STAFFS.

There are still some, chiefly among the less large hospitals, which have not yet decided to make their own arrangements for treating their staffs and employees under the Insurance Act, but many who hesitated to contract out earlier have felt their objections removed or modified by the issue of the revised Form 43*b*. The institutions which have contracted out have decided either to appoint one of their resident medical staff as the panel for the institution in question, or, in the case of the largest institutions, to arrange with the local Insurance Committee for the treatment of their nursing and domestic staffs through the medical and surgical staff as a whole. The latter method is natural to an institution with perhaps several hundred beds, since the staff would be familiar with those who attended them, and treatment in the wards, where necessary, would be easy to arrange; while, conversely, if any nurse or servant were free to make her or his own arrangement for attendance, there would be a possibility that anomalies both in treatment and in discipline might arise, the occasion of which would be much better avoided. Where this idea has been acted upon the names of all the medical staff have been submitted to the local Committee, as those of medical men who are prepared to be responsible for the treatment of the household staff, provided they were allowed to contract out. Thus, free choice of doctor to the hospital nursing and domestic staff was given as far as practicable. Such arrangements also lead to the question arising as to the purpose to which the 9s. per head received in connection with this contracting out should be put. The institution can hardly be regarded as entitled to it, and naturally the honorary medical staff have not wished to pocket it themselves. A *modus vivendi* has sometimes been found in devoting the money to enlarging the institution's medical library.

### THE LONDON MENTAL DEFICIENCY COMMITTEE

The new Mental Hospitals and Mental Deficiency Committee of the London County Council held its first meeting last week, when Mr. Cyril Cobb was elected chairman for the coming year. Mr. Cobb was chairman of the Council last year, and had previously been chairman of the Education Committee, and the experience gained from which should now be useful. Mr. H. V. Rowe, who is interested in mental hospital work, has been appointed vice-chairman, and Mr. Cobb and Mr. Rowe, together with Miss Evelyn Fox, Mr. W. C. Johnson, and Mr. P. E. Pilditch, have been chosen to serve on an interim sub-committee to consider how to carry out the duties imposed on the local authority by the Mental Deficiency Act. The Hon. Mrs. Herbert Lawrence, a co-opted member of the Committee, has resigned, and Miss Henniker has been appointed in her stead.

### THE TRAVELLING EXPENSES OF SANATORIA PATIENTS.

The trustees of the Wilkinson Sanatorium in Lancashire have been informed by the central tuberculosis officer for the county, Dr. Lissard Cox, that the Lancashire Insurance Committee have decided to pay the travelling expenses of patients on arrival and at their departure from the sanatorium. This is a matter which has caused some heart-searching throughout the country, and the practice of the local Insurance Committees is not, we believe, at present uniform. Yet there are at the present time sufficient difficulties, both technical and in the matter of limited accommodation, in the way of granting sanatorium benefit promptly even to the most needy cases, that to send a permit of admission to a patient without seeing whether he is in a position to pay the cost of travelling is a dreadful example of the misery to which official red tape can lead so easily. The Wilkinson Sanatorium, however, has a temporary motive to keep down the numbers of those in residence owing to the internal redecoration which is still going on, and Dr. Marshall, the hon. medical officer, regrets that the waiting list is growing in consequence. We hope that the full accommodation of forty will soon be available. The hon. secretary, Mr. F. Nightingale, has conveyed the trustees' congratulations to the founder of the sanatorium, Mr. Wilkinson, on his eighty-eighth birthday.

### THE ATTENDANCE OF MEDICAL OFFICERS AT GUARDIANS' MEETINGS.

One of the duties of all Poor-Law medical officers, as laid down by the Local Government Board in the General Consolidated Order of 1847, is to attend any meeting of the Board of Guardians when requested by them to do so. The Poor-Law Institutions Order of 1913 makes it the duty of the workhouse medical officer to attend also any meeting of the house committee or of any other duly appointed committee composed of Guardians when similarly directed. These requirements are necessary when reasonably exercised, but it is obvious that they can be made very oppressive, especially in the case of part-time medical officers, if exercised unreasonably. In the case of the East and West Flegg Union, the Guardians recently passed a resolution requiring all their medical officers to attend all ordinary meetings of the Guardians. The medical officers, however, protested. They stated they were perfectly willing to attend when really required, but that such attendance meant a serious interference with their medical work and should not be asked for unless necessary. After considering this protest, the Guardians rescinded their resolution, and decided to ask the medical officers to attend the quarterly meetings of the Board only. We congratulate both the medical officers and the Guardians upon this common-sense decision, and would commend it to the notice of other medical officers faced with similar unreasonable demands upon their time.

### HOURS AND SALARIES OF PUBLIC OPHTHALMIC MEDICAL OFFICERS.

Dr. J. Pringle, assistant medical officer at White Oak Ophthalmic School, and Mr. F. A. C. Tyrell, F.R.C.S., assistant medical officer at High Wood Ophthalmic School, have been reappointed by the Metropolitan Asylums Board. The Children's Committee reported that two questions have arisen in connection with the renewal of these annual appointments—their hours of attendance, and arrangements for medical attendance for general ailments in their absence. Some years ago the hours of attendance required from these officers were increased from three to four consecutive hours daily, and the remuneration increased from £150 to £200 per annum, plus a season ticket from London. In practice there has been considerable difficulty in securing the four hours' attendance, and the ophthalmic surgeon agreed that it was highly improbable that any medical officers of the type required, who are wanted for part time only, would be found to give so large a part of the day to the work as was required—viz., four hours' attendance in addition to the time involved in travelling to and from the schools. The salary of £150 per annum was fixed many years ago, and since that time the rates of remuneration of medical officers in the public service have advanced considerably. The Committee was of opinion that the appointments should in future be made at the existing rate, and that the officers be required to perform the ophthalmic work required by the surgeon, with a minimum attendance of three hours daily. It was decided to secure the services of a local medical practitioner to attend to any urgent work arising during the absence of the assistant medical officers.

### THE INFIRMARY MOVEMENT IN WALES.

Speaking at the opening last week of the new infirmary in connection with the Carnarvon Union, which provides accommodation for sixty patients, Mr. H. R. Williams, Local Government Board Inspector, said that within a few years twenty well-equipped infirmaries would be scattered over the Principality, providing more efficient and humane treatment of the sick poor. A new era, in fact, began this month with the passing away of the names "workhouse" and "pauper," and, the inspector added, "I hope the effect will be to remove much of the prejudice now felt against the Poor Law." Coming from the lips of such an official these words indeed mark not the prospect but the accomplishment of a revolution in the aims and methods of the Poor Law, which is worth recording as only the latest illustration of constitutional, we might almost say, tacit, revolution so dear to the English, and on this occasion at least to the Welsh, character. The new infirmary has been designed by Mr. R. L. Jones, an architect of Carnarvon, and built at a total cost of £9,687. The design furnishes men's and women's wards for sixteen beds each, men's and women's consumptive wards for eight beds each, four isolation wards for each sex, and a lying-in ward with four beds. The medical officer is Dr. Robert Parry.

### GOVERNMENT BY GENERAL COMMITTEE.

The governors of Yarmouth Hospital have held a meeting at which various alterations in the rules have been carried out to effect a less cumbrous method of administration. Hitherto, as the Rev. G. McLuckie pointed out, the entire business of the hospital has been conducted by the whole of the general committee, which has met once a week to transact not merely the necessary routine detail, but also to decide questions of policy and principle. As a result the detail has taken up so much time that when important matters have arisen there has been either no time, or an inadequate amount, in which to discuss them. He therefore moved that all questions of detail, such as checking the accounts and routine matters, be dealt with by a small house committee, on which no member should serve more than four months, so that he hoped that every member of the general committee would pass through the house committee in turn, and thus gain valuable practical experience. The four months' period would prevent the management from falling into the hands of a small section of the general committee, and would lead to greater efficiency. The proposal was carried. It is evident from this that the hospital has outgrown its constitution, and, indeed, it will surprise many institutional workers that a hospital with seventy-two beds, like that at Yarmouth, has been able to do without a house committee for so long.

### A PHARMACIST'S PATH TO FAME.

At last appears the proposed Act of Parliament which is to attempt to remove the anomalies under which dispensing is carried on in this country, an exposure of which was contained in The Hospital, August 31, 1912. The Bill was introduced by Mr. W. S. Glyn-Jones, M.P., a politician who has travelled to fame along an unusual path. It is not a great many years since Mr. Glyn-Jones was dispensing in a doctor's surgery, and fewer since he was conducting a chemist's shop in East London. From this to a seat on the Pharmaceutical Society's Council, to the secretaryship and foundation of the Proprietary Articles Trading Association and the Chemists' Defence Association, thence to a study of the law and emergence as a barrister, finally led him to the House of Commons, where he played some part in moulding the Insurance Act. We discuss the Bill in a leading article this week, merely noting here that it was talked out.

### THE NEW SANATORIUM AT DERBY.

The new sanatorium pavilion, which has been opened at Little Chester by Alderman Dr. R. Laurie, J.P., provides accommodation for twenty-six beds, exclusive of those which can be placed on the verandah, and has been built at a cost of £3,570, of which two-thirds will be provided by the Treasury. Designed by Mr. C. E. Stafford, borough surveyor's office, the new unit comprises a dining-room partitioned for the two sexes, a room for drying clothes, a ward kitchen, two separation wards, bath and lavatories. The doorways are divided into four sections, any of which can be opened as required, according to "the stable" type, while the floor is of "sano." A tuberculosis dispensary has also been opened. Dr. Laurie reminded those present that the voluntary notification of consumption had prevailed at Derby since 1900, when his suggestion for its adoption after the International Tuberculosis Congress of that year had been approved by the local medical practitioners. As a result of this and other activities, he added, when sanatorium benefit was introduced they were fairly well equipped, and all the patients recommended by the Insurance Committee, sooner or later, had been dealt with. How many other towns could make the same boast?

### THE DOCTOR'S WIFE.

A few days only have to elapse before April 18, the date fixed in advance for testing the possibility of forming an Association of Doctors' Wives for the purpose of securing on co-operative lines a free service and purchasing agency. We print on page 52 the coupon which must be filled in and signed by every would-be member of the proposed Association, the success of which largely depends upon 1,000 members joining by April 18. The letters which continue to reach us, and the signatures which continue to come in, only show how the interest awakened by the scheme is growing. The remaining time limit being short, it is essential that every woman, whether a doctor's wife or a woman doctor (since the latter class promptly expressed a desire to be eligible for membership), should make the proposal known to all whom she thinks likely to be interested. Every member who becomes in this sense a recruiting agent of the proposed Association will thus secure that her own enterprise will bear double fruit. To those who are not yet in possession of the facts, and who still desire further information than the coupon on page 52 supplies, we may remind that the original article, which appeared in The Hospital of March 21, has been reprinted and is obtainable on application to "Co-operation," c/o The Scientific Press, 28 Southampton Street, Strand, W.C.

### EASTER AND THE DRUG MARKET.

In view of the near approach of the Easter holidays business in the drug market has been much quieter. Buyers of cod-liver oil still appear to be reluctant to make purchases, although prices can now be regarded as not very much higher than normal figures; the results of the fishing continue to be remarkably good, and it would not be surprising if the yield of oil at the end of the season is not far below the yield in the bountiful season of 1912. There would seem to be no immediate need to secure supplies, and among those who follow the market carefully there are some who predict that prices will be lower before long. Citric acid still tends upwards in price, and there seems to be little likelihood of a decline in value in the near future. Menthol is in plentiful supply and prices have a lower tendency; the present figure, however, is not high, and those who are in need of a supply would probably not gain much by delaying their purchases. As was expected, the price of santonin has been advanced. There is little change in the position of essence of lemon, but the general opinion is that prices will be lower. The value of opium is not quite so firmly maintained, but the future is uncertain. The demand for eucalyptus oil is very quiet, and as the supply is plentiful it does not seem likely that any change in price would be in an upward direction. The value of belladonna root is well maintained. The price of atropine sulphate has been advanced.

